# Generation of a human airway epithelium derived basal cell line with multipotent differentiation capacity

**DOI:** 10.1186/1465-9921-14-135

**Published:** 2013-12-03

**Authors:** Matthew S Walters, Kazunori Gomi, Beth Ashbridge, Malcolm A S Moore, Vanessa Arbelaez, Jonna Heldrich, Bi-Sen Ding, Shahin Rafii, Michelle R Staudt, Ronald G Crystal

**Affiliations:** 1 Department of Genetic Medicine, Weill Cornell Medical College, New York NY, USA; 2 Cell Biology Program, Memorial Sloan-Kettering Cancer Center, New York NY, USA

**Keywords:** Airway, Basal cell, Immortalized, hTERT, Progenitor, Differentiation

## Abstract

**Background:**

As the multipotent progenitor population of the airway epithelium, human airway basal cells (BC) replenish the specialized differentiated cell populations of the mucociliated airway epithelium during physiological turnover and repair. Cultured primary BC divide a limited number of times before entering a state of replicative senescence, preventing the establishment of long-term replicating cultures of airway BC that maintain their original phenotype.

**Methods:**

To generate an immortalized human airway BC cell line, primary human airway BC obtained by brushing the airway epithelium of healthy nonsmokers were infected with a retrovirus expressing human telomerase (hTERT). The resulting immortalized cell line was then characterized under non-differentiating and differentiating air-liquid interface (ALI) culture conditions using ELISA, TaqMan quantitative PCR, Western analysis, and immunofluorescent and immunohistochemical staining analysis for cell type specific markers. In addition, the ability of the cell line to respond to environmental stimuli under differentiating ALI culture was assessed.

**Results:**

We successfully generated an immortalized human airway BC cell line termed BCi-NS1 via expression of hTERT. A single cell derived clone from the parental BCi-NS1 cells, BCi-NS1.1, retains characteristics of the original primary cells for over 40 passages and demonstrates a multipotent differentiation capacity into secretory (MUC5AC, MUC5B), goblet (TFF3), Clara (CC10) and ciliated (DNAI1, FOXJ1) cells on ALI culture. The cells can respond to external stimuli such as IL-13, resulting in alteration of the normal differentiation process.

**Conclusion:**

Development of immortalized human airway BC that retain multipotent differentiation capacity over long-term culture should be useful in understanding the biology of BC, the response of BC to environmental stress, and as a target for assessment of pharmacologic agents.

## Introduction

The human airway epithelium, a complex pseudostratified multicellular layer that lines the bronchial tree, is comprised of ciliated, secretory, intermediate/undifferentiated and basal cells (BC) [1-3]. The BC, a proliferating population of cells that reside in close proximity to the basement membrane, function as stem/progenitor cells of both the mouse and human airway epithelium and are capable of differentiating into the other specialized cell types during normal epithelial turnover and repair [4-18]. In response to stress, such as cigarette smoke or inflammatory stimuli, this differentiation process is altered, resulting in a disordered airway epithelium [19-26]. Understanding the mechanisms by which the differentiation capacity of the BC is regulated and how the cells respond to specific environmental stimuli is central to the understanding of diseases characterized by airway epithelial remodeling such as chronic obstructive pulmonary disease (COPD) and asthma, and the biology of the malignant transformation of the epithelium into bronchogenic carcinoma [3,8,26-30].

The ability to have long-term replicating cultures of airway BC that maintain features of their original phenotype would provide a convenient means to study the mechanisms that regulate BC differentiation, assess the response of BC to environmental stress and screen drugs targeted toward suppressing or activating specific pathways. The challenge in studying these processes is that, like all normal somatic cells, cultured primary human airway BC divide only a limited number of times before entering a state of replicative senescence. The short lifespan of primary BC cultures, coupled with biological changes that occur as cells reach senescence, limits the experimental scope for which each culture can be utilized [31,32]. To address this problem, various different strategies have been employed. For instance, recent studies have demonstrated that primary human epithelial cells (including human airway epithelium) can be propagated indefinitely *in vitro* when co-cultured with irradiated fibroblast feeder cells and a Rho kinase inhibitor [33,34]. Prior studies have demonstrated that long term cultures of human bronchial epithelium obtained from bronchial derived donor material can be established using a number of different methods, including adenovirus-SV40 hybrid virus; plasmid containing a replication defective SV40 virus genome; and plasmid or retroviral gene transfer-mediated delivery of viral oncoproteins (HPV-16 E6 and E7, or SV40 T-antigen) alone or in combination with the catalytic subunit of human telomerase reverse transcriptase (hTERT) [35-41]. Alternative strategies to viral oncoproteins have used retroviral gene transfer-mediated expression of hTERT alone or together with cyclin dependent kinase 4 (CDK4) or B-cell Moloney murine leukemia retrovirus-specific integration site 1 (Bmi-1). Cells produced by these strategies have an extended life span far beyond normal senescence and retain characteristics of the primary cells [42-46].

Based on these observations, and utilizing methodology in our laboratory to culture pure populations of human airway BC from the airway epithelium obtained by brushing the airway epithelium of healthy nonsmokers, we have successfully immortalized a human airway BC cell line derived from a healthy nonsmoker via retrovirus-mediated expression of hTERT. The resulting cell line, termed basal cell immortalized-nonsmoker 1 (BCi-NS1), and a clonal population of the parental cells (BCi-NS1.1) retain characteristics of the original primary cells, maintain a multipotent differentiation capacity for over 40 passages and respond to external stimuli to alter the normal differentiation process.

## Methods

### Sampling airway epithelium and culture of primary human airway basal cells

Under a protocol approved by the Weill Cornell Medical College Institutional Review Board, healthy nonsmokers were recruited for this study. The subjects were confirmed to be nonsmokers by urine levels of nicotine (<2 ng/ml) and cotinine (<5 ng/ml) with normal pulmonary function tests and chest X-ray. Following written informed consent, flexible bronchoscopy was used to collect large airway epithelial cells by brushing the epithelium [47-49]. Basal cells (BC) were subsequently purified from the total airway epithelium brushings by trypsinization of the cells and selective culturing of BC on T25 cm^2^ plastic tissue culture flasks as previously described [4,50]. The airway epithelial cells collected by brushing were pelleted by centrifugation (250 × g, 5 min) and disaggregated by resuspension in 0.05% trypsin-ethylenediaminetetraacetic acid (EDTA) for 5 min, at 37°C. Trypsinization was stopped by addition of HEPES buffered saline, (Lonza, Basel, Switzerland) supplemented with 15% fetal bovine serum (FBS; GIBCO-Invitrogen, Carlsbad, CA), and the cells were again pelleted at 250 × g, 5 min. The pellet was resuspended with 5 ml of phosphate buffered saline, pH 7.4 (PBS), at 23°C, then centrifuged at 250 × g, 5 min. Following centrifugation, the PBS was removed and the cells resuspended in 5 ml of Bronchial Epithelial Growth Media (BEGM, Lonza, CA) and 5 × 10^5^ cells plated in T25 flasks in 5 ml of BEGM and maintained in a humidified atmosphere of 5% CO_2_ at 37°C. The next day, unattached cells were removed by changing the medium and thereafter, every 2 days. Following 7–8 days of culture, when the cells were 70% confluent, they were characterized by immunohistochemical staining of tryspinized cytopreps using cell type specific markers as being >99% BC (KRT5^+^, TP63^+^, CD151^+^, β-tubulin IV^-^, MUC5AC^-^, TFF3^-^, CC10^-^, chromogranin A^-^ and N-cadherin^-^) and when put on air-liquid interface (ALI) culture, were capable of differentiating into a mucociliary epithelium [4]. To passage the cells, the primary BC were seeded at a cell density of 3000 cells/cm^2^ in BEGM. The following day, the media was replaced with fresh BEGM and thereafter, every 2 days.

### Generation of an hTERT expressing retrovirus

Human telomerase (hTERT) was PCR amplified with forward (5′-CGATCGATGCCACCATGCCGCGAGCTCCCCGTTGCCGAG-3′) and reverse (5′- GGTACGTATCAGTCCAGGATGGTCTTGAAGTCTG-3′) primers and cloned into the TOPO® TA subcloning vector (Invitrogen, Carlsbad, CA) using the manufacturer’s protocol. The hTERT insert was then subcloned into the pBABE-puromycin retroviral vector [51] via the EcoRI restriction site. The resulting plasmid pBABE-puro-hTERT was sequenced to verify the correct orientation and integrity of the hTERT open reading frame. Recombinant replication deficient retroviruses were generated by transient co-transfection of 293A cells with pBABE-puro-hTERT and the packaging plasmids pGAG-Pol and pMD.G (VSVg envelope). The virus containing media was collected at 24 hr intervals with replacement of fresh media at each time point. At 72 hr post transfection, the media was harvested and pooled with previous time points for subsequent virus purification by standard methods.

### Immortalization of human airway basal cells

Cultured primary airway BC from a 42 yr old Hispanic, male, healthy nonsmoker were immortalized using retrovirus-mediated expression of hTERT. The primary BC were passaged once and subsequently seeded at a density of 10^5^/cm^2^ into each well of a 6-well plate in BEGM. The following day, the media was replaced and 24 hr later the cells infected overnight with the retrovirus expressing hTERT and the puromycin resistance selection marker in the presence of polybrene (2 μg/ml). The next day, the cells were washed with PBS and then incubated for 3 days in BEGM. After 3 days, selection media (BEGM containing 0.5 μg/ml of puromycin) was added for 10 days. As a control to demonstrate the activity and killing effect of the puromycin, uninfected BC (non-puromycin resistant) were treated simultaneously. Once selection of resistant cells was complete, the cells were cultured in BEGM (minus puromycin) in the same way as primary BC. This parental immortalized cell line was termed basal cell immortalized-nonsmoker 1 (BCi-NS1). A clonal population of immortalized BC (BCi-NS1.1) was generated by serial dilution of the parental cells and subsequent expansion of isolated single cell colonies. Based on the findings of previous studies that immortalization of primary human airway epithelium with hTERT alone is either inefficient or requires additional genes to extend the lifespan of cells beyond 30 passages (Additional file 1: Table S1), we used the criteria of lifespans greater than 40 passages to deem the cells immortalized. The immortalized BC were cultured in an identical manner to primary BC and seeded at a cell density of 3000 cells/cm^2^ in BEGM for all experiments unless stated otherwise.

### Measurement of telomerase activity

Telomerase activity was measured by the PCR-based Telomeric Repeat Amplification Protocol (TRAP) according to the manufacturer’s instructions (TRAPeze® Telomerase Detection Kit; Cat# S7700; Millipore, Billerica, MA). Briefly, cell lysates from the parental primary BC and immortalized BCi-NS1 cells were generated and an equal amount of total protein (2 ug) added to each reaction. A telomerase positive HeLa cell extract provided with the TRAPeze® kit was used as a positive control and heat inactivation of the same extract was used as a negative control. Following PCR amplification, the Cy5-labeled products were resolved on a polyacrylamide gel and visualized using a Molecular Dynamics Typhoon Trio + (GE Healthcare Biosciences, Pittsburgh, PA) to determine telomerase activity.

### Characterization of human airway basal cells

The primary donor, immortalized parental BC and the BCi-NS1.1 clone were characterized by expression of cell type specific markers using previously described methods [4,50]. Cytospin slides of trypsinized cultured BC were prepared for characterization by immunohistochemistry (described below), using the following cell-type specific markers: KRT5 (basal cell); TP63 (basal cell); CD151 (basal cell); β-tubulin IV (ciliated cell); MUC5AC (secretory cell); TFF3 (goblet cell); CC10 (Clara cell); chromogranin A (neuroendocrine cell); and N-cadherin (mesenchymal cell). Only cultures >99% positive for basal cell markers and negative for other cell types were used in this study. Karyotype analysis of the immortalized cells was performed at the Molecular Cytogenetics-Core Facility at Memorial Sloan-Kettering Cancer Center using established protocols (http://www.mskcc.org/research/molecular-cytogenetics). At least 17 metaphase spreads were analyzed for each passage of BCi-NS1.1 cells.

### Western analysis

Cells were harvested and lysed in radioimmunoprecipitation lysis (RIPA) buffer (Sigma, St. Louis, MO) containing complete protease inhibitor cocktail (Roche, Mannheim, Germany) and halt phosphatase inhibitor cocktail (Pierce, Rockford, IL). The protein concentration was then quantified using the Bradford Assay and an appropriate volume of 4X NuPAGE LDS sample buffer (Invitrogen) containing 200 mM dithiothreitol (DTT) added to each sample. The cellular lysates were then boiled for 10 min and equal amounts of total protein for each sample analyzed using NuPAGE 4–12% Bis–Tris gradient gels (Invitrogen) and subsequently transferred onto nitrocellulose membranes with a Bio-Rad semidry apparatus before Western analysis. The membranes were then blocked overnight at 4°C in 4% blocking reagent (Nonfat milk) made in PBS containing 0.1% Tween-20 (PBST). After blocking the membranes overnight, immobilized proteins were reacted with the following cell-type-specific antibodies in 4% blocking reagent for 1 hr, 25°C with shaking: KRT5 (basal cell; 1/5000; PA1-37974; Thermo Scientific, Rockford, IL); TP63 (basal cell; 1/3000; sc-8431; Santa Cruz Biotechnology, Inc., Santa Cruz, CA); MUC5AC (secretory cell; 1/500; VP-M657; Vector Laboratories, Burlingame, CA); CC10 (Clara cell; 1/10000; RD181022220; BioVendor LLC, Candler, NC); DNAI1 (ciliated; 1/2000; HPA021649; Sigma); FOXJ1 (ciliated; 1/1000; 14–9965; eBioscience, San Diego, CA); and GAPDH (loading control; 1/10000; sc-32233; Santa Cruz Biotechnology, Inc). Following the primary antibody incubation, membranes were washed three times for 5 min each with PBST, followed by incubation with an anti-rabbit or anti-mouse antibody conjugated to horseradish peroxidase in 4% blocking reagent for 1 hr at room temperature with shaking. On completion of the secondary antibody incubation, the membranes were then washed again three times for 5 min with PBST and twice with PBS, and antibodies visualized after the addition of ECL Western analysis detection reagents (GE Healthcare Biosciences) by exposure to X-ray film. For Western analysis of ALI experiments, the cells were lysed directly in ALI Transwell inserts using 100 μl of 1 × NuPAGE LDS sample buffer (Invitrogen) diluted in radioimmunoprecipitation lysis (RIPA) buffer (Sigma) containing complete protease inhibitor cocktail (Roche), halt phosphatase inhibitor cocktail (Pierce) and 50 mM dithiothreitol (DTT). The lysates were then boiled for 10 min and equal volumes of each sample analyzed using NuPAGE 4–12% Bis–Tris gradient gels (Invitrogen) and subsequently processed as described above.

### Immunohistochemistry

For characterization of BC cultures by immunohistochemistry, cytospin slides of trypsinized cells were prepared, and subsequently fixed in 4% paraformaldehyde for 15 min. An antigen recovery step was carried out by steaming the samples for 15 min in citrate buffer solution (Thermo Scientific), followed by cooling at 23°C for 20 min to enhance staining. Endogenous peroxidase activity was quenched using 0.3% H_2_O_2_ for 30 min, followed by incubation for 20 min with normal serum matched to the secondary antibody to reduce background staining. Samples were incubated overnight at 4°C with the following primary antibodies: KRT5 (basal cell; 1/500; PA1-37974; Thermo Scientific); TP63 (basal cell; 1/200; sc-8431; Santa Cruz Biotechnology, Inc.); CD151 (basal cell; 1/200; NCL-CD151; Leica Microsystems, Inc., Bannockburn, IL); β-tubulin IV (ciliated cell; 1/1000; MU178-UC; Biogenex, San Ramon, CA); MUC5AC (secretory cell; 1/70; VP-M657; Vector Laboratories); TFF3 (goblet cell; 1/200; 2816–1; Epitomics, Burlingame, CA); CC10 (Clara cell; 1/10000; RD181022220; BioVendor LLC); chromogranin A (neuroendocrine cell; 1/5000; MS-382-P; Thermo Scientific); and N-cadherin (mesenchymal cell; 1/500; 33–3900; Invitrogen). Isotype-matched IgG (Jackson ImmunoResearch Laboratories Inc., West Grove, PA) was used as a negative control. The Vectastain Elite ABC kit and AEC substrate kit (Dako North America Inc., Carpinteria, CA) were used to visualize antibody binding and slides were counterstained with Mayer’s hematoxylin (Polysciences Inc., Warrington, PA) and mounted using faramount mounting medium (Dako North America Inc.). Images were acquired using a Nikon Microphot microscope with a Plan 60 × N.A. 0.70 oil immersion objective lens and an Olympus DP70 CCD camera. For all experiments, appropriate positive control tissues were used to confirm specificity and positive staining for each antibody.

### ELISA

The secretion of VEGFA by primary BC and BCi-NS1.1 cells was assessed by ELISA (R&D Biosystems, Minneapolis, MN). For each cell type, 10^5^ cells were seeded into each well of a 12-well plate in duplicate in BEGM growth media. The next day, the cells were washed one time with PBS and incubated for 24 hr in 1 ml BEGM. Following incubation, the media was removed and then centrifuged at 250 × g, 5 min to pellet cellular debris. The supernatant was then analyzed by ELISA using the manufacturer’s instructions. Growth media (BEGM) not exposed to BC and containing no VEGFA was used as a negative control. For each sample, the final cell numbers following media harvest were counted and used to calculate the levels of secreted VEGFA per cell. Final calculations of secreted VEGFA for each sample represent an average of the duplicate reactions. For primary BC, each independent culture was passaged a maximum of three times before ELISA analysis.

### Endothelial Co-culture proliferation assays

Co-culture proliferation assays were used to assess the ability of endothelial cells to support primary BC and BCi-NS1.1 proliferation in cytokine- and serum-free conditions as previously described [50]. Briefly, HUVEC-Akt cells (5 × 10^4^) were seeded into each well of a 12-well plate in HUVEC growth media. The next day the media was removed and the cells washed twice with PBS, followed by seeding 2 × 10^4^ primary BC or BCi-NS1.1 cells into each well in BEGM growth media. The next day (termed day 0) the media was removed and the cells washed twice with PBS before addition of cytokine- and serum-free BEBM media. The cells were then incubated and subsequently harvested and counted at the desired time points. As a control, primary BC or BCi-NS1.1 cells were seeded into wells containing no HUVEC-Akt controls and subsequently treated in an identical manner to those in co-culture. At the desired time points, cells were trypsinized and total cell numbers were measured with a hemocytometer and the viability assessed by counting of trypan blue dye-excluded cells. The population of GFP-labeled HUVEC-Akt cells in the harvested sample was determined as the GFP^+^VE-cadherin^+^ population by flow cytometric analysis, and the GFP^-^VE-cadherin^-^ population quantified as expanded primary BC or BCi-NS1.1 cells. For primary BC, each independent culture was passaged one time to obtain sufficient cell material before study in co-culture.

### Air-liquid interface culture

To investigate the differentiation capacity of the immortalized BC, the cells were grown on air-liquid interface (ALI) cultures using established methods [4,50]. The immortalized BC were trypsinized and then seeded at a density of 4.5 ×10^5^ cells/cm^2^ onto a transwell insert (0.4 μm size pore; Corning Incorporated, NY) coated with human type IV collagen (Sigma) in media consisting of a 1:1 mixture of DMEM (Cellgro, Manassas, VA) and Ham’s F-12 Nutrient Mix (Invitrogen) supplemented with 100 U/ml penicillin, 5% fetal bovine serum 100 μg/ml streptomycin, 0.1% gentamycin and 0.5% amphotericin B. Following overnight incubation, the media was replaced with 1:1 DMEM/Ham’s F12 (including antibiotics described above) supplemented with 2% of the serum substitute Ultroser G (BioSerpa S.A., Cergy-Saint-Christophe, France). Two days post seeding, the media was removed from the upper chamber to expose the apical surface to air and establish the ALI (referred to as ALI day 0). The ALI cultures were then grown at 37°C, 8% CO_2_, with fresh media changes every 2 to 3 days. Following 5 days on ALI, the CO_2_ levels were reduced to 5% until harvest of the cultures at ALI day 28. The transepithelial resistance (TER) was measured at each media change using a Millicell-ERS epithelial ohm meter (Millipore, Bedford, MA) and results plotted over the course of time. For each time point and condition, the resistance of 3 replicate ALI wells was assessed. For experiments investigating the effect of IL-13 stimulation on differentiation of the immortalized BC, the standard ALI method was employed and 5 ng/ml of IL-13 (Peprotech, Rocky Hill, NJ) added at ALI day 10 to the basolateral media. The IL-13 stimulation was maintained until ALI day 28 with fresh IL-13 added at each media change. Experiments studying the differentiation of immortalized BC were performed with cells grown between passages 6 to 43. For primary BC, each independent culture was passaged one time to obtain sufficient cell material before study on ALI. Histological analysis of ALI day 28 cultures for both primary BC and BCi-NS1.1 cells was performed via staining of paraffin embedded ALI sections. Briefly, ALI day 28 transwell inserts were fixed directly with 4% paraformaldehyde in PBS for 20 min and then shipped in 70% ethanol to Histoserv, Inc (Germantown, MD) whereupon the membrane was removed from the transwell insert and subsequently dehydrated through graded alcohols, cleared in xylene and then infiltrated with low melt temperature paraffin. Following processing, the circular membrane was bisected and both halves embedded on the cut edge in the same paraffin block. Five micron sections were then generated and mounted on slides for subsequent analysis.

### Immunofluorescence

Differentiation of primary and immortalized BC on ALI culture was confirmed using immunofluorescent staining of paraffin embedded cross-sections or directly by top-staining of the ALI membrane. For analysis of paraffin embedded sections, the samples were first cleaned in xylene and rehydrated with graded ethanol. To unmask the antigens, samples were steamed for 15 min in citrate buffer solution (Thermo Scientific), followed by cooling at 23°C for 20 min, then blocked for 30 min with normal serum to reduce background staining. For direct top-staining, the ALI membranes were fixed directly with 4% paraformaldehyde in PBS for 20 min and then permeabilized with 0.1% triton X-100 in PBS, followed by blocking with normal serum. The samples were then treated and stained with the following primary antibodies: KRT5 (basal cell; 1/100; PA1-37974; Thermo Scientific); β-tubulin IV (ciliated cell; 1/100; MU178-UC; Biogenex); CC10 (Clara cell; 1/200; RD181022220; BioVendor LLC); MUC5AC (secretory cell; 1/50; VP-M657; Vector Laboratories); and TFF3 (goblet cell; 1/100; 2816–1; Epitomics) for 60 min at 23°C. Isotype matched IgG (Jackson Immunoresearch Laboratories) was the negative control. To visualize the antibody binding, Alexa Fluor® 488 Goat Anti-Mouse IgG (A-11029; Invitrogen) and Alexa Fluor® 546 Goat Anti-Rabbit IgG (A-11035; Invitrogen) labeled secondary antibodies were used. The cells were counterstained with DAPI to identify cell nuclei and subsequently mounted using ProLong® Gold antifade reagent (Invitrogen). For top-staining, the ALI membrane was cut from the well before mounting. Immunofluorescent microscopy was performed using a Zeiss Axioplan body microscope with either a 20 × or 40 × lens. The images were captured with a Zeiss hrM (high resolution monochrome) camera.

### Quantification of differentiation

To quantify the differentiation capacity of primary BC and immortalized BC following ALI culture, paraffin-embedded sections were stained with Alcian blue/Nuclear Fast Red Kernechtrot Reagents (Cat# K066; Poly Scientific R&D Corp., Bay Shore, NY) to determine the percentage of ciliated and secretory cells. A minimum of 10 images equally distributed between both ends of the sectioned membrane were acquired using a Nikon Microphot microscope with a Plan 40 × N.A. 0.70 objective lens and an Olympus DP70 CCD camera. Ciliated cells were determined by morphology and the number of secretory cells by Alcian blue staining. For each sectioned membrane, a minimum of 250 total cells were counted.

### TaqMan gene expression

Total RNA was extracted using a modified version of the TRIzol method (Invitrogen), in which RNA is purified directly from the aqueous phase using the Rneasy MinElute RNA purification kit (Qiagen, Valencia, CA). RNA concentration was determined using a NanoDrop ND-1000 spectrophotometer (NanoDrop Technologies, Wilmington, DE) and cDNA synthesized from 1 μg of total RNA using TaqMan Reverse Transcription Reagents (Applied Biosystems, Foster City, CA). All samples were then analyzed in duplicate at two different cDNA dilutions (1:10 and 1:100) to ensure amplification efficiency of the reference gene and gene of interest are consistent. All reactions were run on an Applied Biosystems Sequence Detection System 7500 and relative expression levels determined using the dCt method with 18S ribosomal RNA as the endogenous control. Premade TaqMan Gene Expression Assays were obtained from Applied Biosystems: CLDN3 (Hs00265816_s1); CLDN8 (Hs00273282_s1); KRT5 (Hs00361185_m1); TP63 (Hs00978343_m1); MUC5AC (Hs01365616_m1); MUC5B (Hs00861588_m1); CC10 (SCGB1A1, Hs00171092_m1); TFF3 (Hs00902278_m1); DNAI1 (Hs00201755_m1); FOXJ1 (Hs00230964_m1); and 18S rRNA (TaqMan® Ribosomal RNA Control, VIC, #4308329). For each time point and condition, gene expression levels in a minimum of 2 replicate ALI wells were assessed.

### Scratch and wound healing assay

Air-liquid interface cultures of immortalized BCi-NS1.1 cells were set up as described above. At ALI day 0, following exposure of the apical surface to air, a sterile P10 pipette tip was used to scratch across the epithelial surface to induce injury. The apical surface was then washed once with PBS to remove the unattached cells from the injured area, and the cells were subsequently incubated under standard ALI culture conditions to assess the ability of the cells to repair the injured region. Images were acquired at the time of injury (t = 0) and following repair to document the repair process. The procedure was repeated at additional time points during ALI culture (ALI day 7, 14, 21 and 28).

### Statistical analysis

All data included in this study are presented as the average ± standard error. Statistical comparisons were calculated using an unpaired, 2-tailed Student’s t test with unequal variance. A p value <0.05 was considered significant.

## Results

### Immortalization of primary human airway basal cells

**Figure 1:**
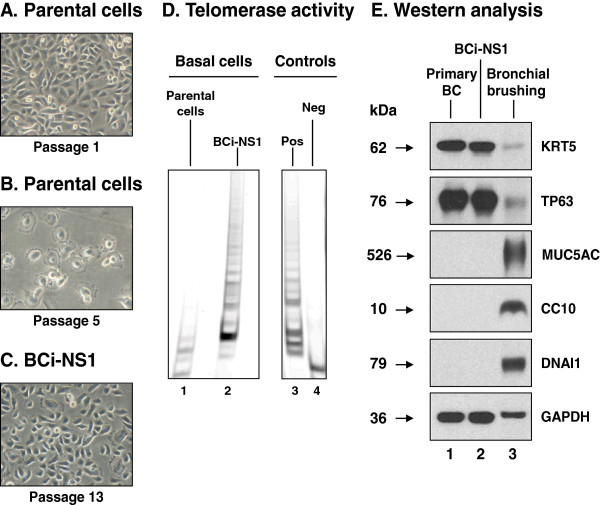
**Isolation and generation of the immortalized human airway basal cell line BCi-NS1. A**. Morphology of the parental primary basal cells at passage 1. **B**. Morphology of the primary basal cells following senescence at passage 5 of culture. **C**. Morphology of immortalized basal cells (BCi-NS1) following passage 13 of culture. **D**. Telomerase activity of immortalized basal cells (TRAP assay). Lane 1 – Parental primary basal cells; lane 2 – immortalized basal cells BCi-NS1; lane 3 – positive control (Pos; telomerase positive HeLa cell extract); lane 4 – negative control (Neg; heat-inactivated telomerase positive HeLa cell extract). **E**. Characterization of immortalized basal cells by Western analysis of cell type specific markers. Lane 1 – Primary basal cells; lane 2 – BCi-NS1 immortalized basal cells; and lane 3 – bronchial brushing. For all cells, shown is the expression of basal cells markers (KRT5, TP63), secretory cell marker (MUC5AC), Clara cell marker (CC10) and ciliated cell marker (DNAI1). GAPDH was used as a loading control.

To establish an immortalized human airway basal cell (BC) cell line, primary airway BC were isolated with selective culture methods from the large airway epithelium of a male healthy nonsmoker. The cells displayed a healthy cobble stone morphology typical for airway BC (Figure 1A). In contrast to the parental primary BC which reached senescence following 5 passages (Figure 1B), the hTERT modified, puromycin resistant populations of BCi-NS1 cells continued to proliferate and grow beyond 40 passages while retaining a healthy morphology, demonstrating that ectopic expression of hTERT was sufficient to extend the lifespan of primary BC (Figure 1C). A telomeric repeat amplification protocol (TRAP) assay confirmed telomerase activity within the BCi-NS1 cells. Analysis of cell lysates from the primary BC demonstrated no hTERT activity and appeared identical to the negative control (Figure 1D, lanes 1, 4). In contrast, the cell lysates from the BCi-NS1 cells were positive for hTERT activity resulting in large quantities of telomeric repeat containing PCR products of increasing length similar to those present in the positive control (Figure 1D, lanes 2, 3).

To further characterize the BCi-NS1 cells and determine if they retain characteristics of primary BC, Western analysis was carried out using antibodies against basal cell (KRT5, TP63); secretory cell (MUC5AC); Clara cell (CC10); and ciliated cell (DNAI1) specific markers (Figure 1E). The basal cell markers were expressed at comparable levels in the primary BC and BCi-NS1 cells but at higher levels relative to the bronchial brushing positive control. In contrast, the differentiated cell markers (MUC5AC, CC10 and DNAI1) were only detected in the bronchial brushing and absent from the primary BC and BCi-NS1 cells. Overall, these data demonstrate that over-expression of hTERT is sufficient to extend the lifespan of primary human airway BC while retaining the morphological characteristics of the primary cells. We assessed the effect of continuous culture on cell viability of the BCi-NS1 cells by calculating the percentage of viable cells using trypan blue exclusion at early and late passages. Comparison of eleven early passages (passage 8–20) *vs* eleven later passages (passage 35–46) showed a significant, but minor, increase (p < 0.01) in cell viability for later (86.5%) *vs* early (80.5%) passages (Additional file 1: Figure S1A). These data suggest that continuous serial culture of the cells selects for a population of cells with increased viability.

### Isolation of a clonal immortalized human airway basal cell line

**Figure 2:**
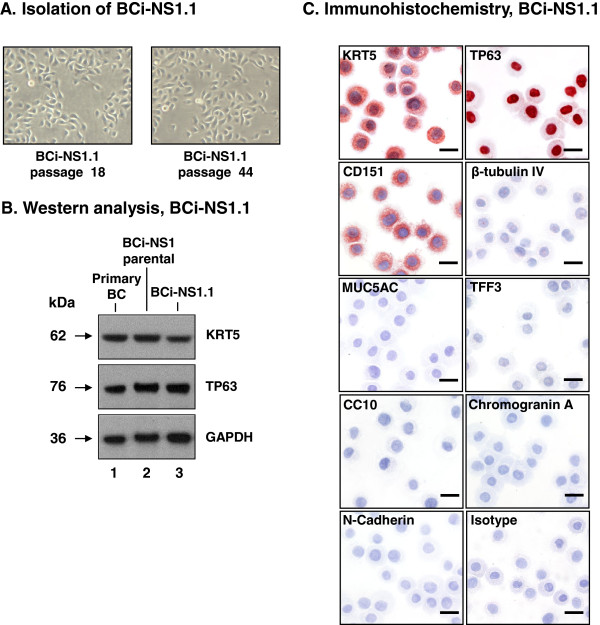
**Isolation of BCi-NS1.1 clone of the BCi-NS1 immortalized human airway basal cell line. A**. Morphology of BCi-NS1.1 clone at passage 18 and passage 44. **B**. Comparison of the parental and clonal immortalized basal cells by Western analysis of cell type specific markers. Lane 1 – Primary basal cells; lane 2 – BCi-NS1 parental immortalized basal cells, passage 24; and lane 3 – BCi-NS1.1 clonal immortalized basal cells, passage 3. For all cells, shown is the expression of the basal cell markers KRT5 and TP63. GAPDH was used as a loading control. **C**. Immunohistochemical characterization of cytopreps of BCi-NS1.1 cells (passage 22) with cell-type specific markers: KRT5 (basal cell); TP63 (basal cell); CD151 (basal cell); β-tubulin IV (ciliated cell); MUC5AC (secretory cell); TFF3 (goblet cell); CC10 (Clara cell); chromogranin A (neuroendocrine cell); N-cadherin (mesenchymal cell) and isotype control. Bar = 20 μm.

In order to fully characterize the functional properties of the BCi-NS1 cells, an individual clone, BCi-NS1.1, was isolated using serial dilution of passage 19 BCi-NS1 cells. Comparison of the parental (BCi-NS1) and clonal (BCi-NS1.1) cells at the morphological level demonstrated both cell types displayed a healthy cobble stone morphology typical for airway BC (Figure 2A compared to Figure 1C). Western analysis using antibodies against basal cell specific markers (KRT5, TP63) revealed that the primary BC and the parental (BCi-NS1) and clonal (BCi-NS1.1) immortalized cells were positive for both markers (Figure 2B). Immunohistochemistry of cytospin slides revealed the BCi-NS1.1 cells were >99% positive for the basal cells markers KRT5, TP63 and CD151, but negative for the differentiated cell markers β-tubulin IV (ciliated cell); MUC5AC (secretory cell); TFF3 (goblet cell); CC10 (Clara cell); chromogranin A (neuroendocrine cell); and N-cadherin (mesenchymal cell) (Figure 2C). Analysis of the BCi-NS1.1 cells at twelve early passages (passage 6–23) *vs* twelve later passages (passage 41–71) showed no significant change (p > 0.4) in cell viability between early (89.6%) and late (91.4%) passages (Additional file 1: Figure S1B).

To determine whether hTERT immortalization of human airway BC induced genetic abnormalities, karyotype analysis was carried out on passage 9 BCi-NS1.1 cells. At passage 9, the majority of the cells had a normal male karyotype (46,XY) with approximately 30% containing trisomy 20. There were isolated single individual cells with a single reciprocal translocation as the sole detectable abnormality [46,XY,t(15;16)(p12;p11) and 46,XY,t(6;11)(~p23;q13 ~ 4)]. The presence of heterogeneity in the BCi-NS1.1 cells suggest that genetic abnormalities resulting from hTERT immortalization arose early in the expansion phase of the isolated clone. To assess the progressiveness of these genetic alterations, we analyzed the karyotype at a later passage. The results demonstrated that by passage 35, trisomy 20 was present in approximately 45% of cells and one of the previous observed translocations [46,XY,t(15;16)(p12;p11)] was present as a minor clone (10%). The increase in frequency of trisomy 20 and the single translocation with long term culture suggest these genetic abnormalities convey a competitive growth advantage in culture. A recent study by Tabach et al. [52] using prostate epithelial cells demonstrated that hTERT induced immortalization resulted in amplification of chromosome 20q13 early in the transformation process. With increasing passage of the cells, the frequency of trisomy 20q13 increased and positively correlated with the increasing proliferation rate of the cells with continuing culture. However, analysis of BCi-NS1.1 cells during early (passage 9–12), middle (passage 46–49) and late (passage 71–74) passages saw no significant difference (p > 0.25) in proliferation rate of the cells at any time point with increasing passage (Additional file 1: Figure S2). Therefore, the proliferative effects of trisomy 20 may be cell type specific.

### BCi-NS1.1 cell retain the capacity of primary airway basal cells to secrete VEGFA and cross-talk with endothelial cells

**Figure 3:**
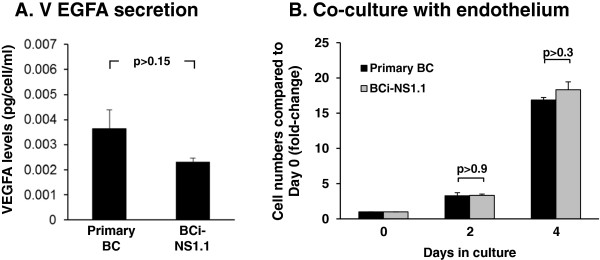
**Secretion of VEGFA by immortalized BCi-NS1.1 cells. A**. VEGFA levels assessed by ELISA in growth media from primary airway basal cell cultures (n = 4) and immortalized BCi-NS1.1 cell cultures (n = 4). Secreted VEGFA was normalized to cell number and calculated as pg/cell/ml. Data shown is the average ± the standard error of n = 4 independent cultures of primary BC and n = 4 independent passages of BC-NS1.1 cells from passage 10 to 14. Statistics were calculated by a 2-tailed Student’s t test. **B**. Endothelial cells support the growth of primary airway basal cells and immortalized BCi-NS1.1 cells in the absence of growth factors. Human primary BC and immortalized BCi-NS1.1 cells were co-cultured with Akt-activated human umbilical vein endothelial cells (HUVEC-Akt) in cytokine- and serum-free conditions. At the desired time points, cells were harvested and the GFP-labeled HUVEC-Akt cells was determined as the GFP^+^VE-cadherin^+^ population by flow cytometric analysis, and the GFP^-^VE-cadherin^-^ population quantified as expanded basal cells. Data shown is the average ± the standard error of n = 3 independent cultures of primary BC and n = 3 independent passages of BC-NS1.1 cells from passage 5 to 26 compared directly in tandem for each independent experiment. Statistics were calculated by a 2-tailed Student’s t test.

To determine whether immortalization had any deleterious effects on cellular function, we assessed whether immortalized BCi-NS1.1 cells retain the ability of primary BC to secrete vascular endothelial growth factor A (VEGFA) and proliferate under cytokine- and serum-free conditions during co-culture with endothelial cells [50]. The secretion of VEGFA by primary BC and BCi-NS1.1 cells was assessed using ELISA (Figure 3A). Growth media exposed to primary BC and BCi-NS1.1 cells for 24 hr was removed and processed as described in Methods. Comparison of four independent cultures of primary BC and four independent passages of BCi-NS1.1 cells revealed primary BC secrete on average 0.0036 pg/cell/ml of VEGFA, whereas BCi-NS1.1 cells secrete on average 0.0023 pg/cell/ml (Figure 3A). There was no statistical significance between these levels of secreted VEGFA (p > 0.15). The levels of secreted VEGFA appeared highly variable between independent primary BC cultures, whereas minimal variation was observed in the BCi-NS1.1 cells between different passages which most likely reflect the clonality of the cell line. We also assessed the ability of the BCi-NS1.1 cells to interact and cross-talk with endothelial cells in a cytokine- and serum-free co-culture system. Primary BC or BCi-NS1.1 cells were co-cultured with HUVEC-Akt cells (in growth factor negative media) and proliferation was quantified every two days. As expected, when cultured alone in the absence of growth factors, primary BC and BCi-NS1.1 failed to proliferate over the course of 4 days (data not shown). However, when co-cultured with HUVEC-Akt cells, at 4 days post-culture, both primary BC and BCi-NS1.1 cell proliferation was observed and the total cell number increased 16.9-fold and 18.3-fold relative to day 0, respectively, for each cell type (Figure 3B). No statistical significance was observed between the endothelial dependent proliferation of primary BC compared to BCi-NS1.1 cells at any time point during co-culture with endothelium (p > 0.9, day 2 and p > 0.3, day 4). Overall, these data demonstrate that immortalization of primary BC with hTERT has minimal deleterious effects on cell function and BCi-NS1.1 cells retain key characteristics of primary BC.

### BCi-NS1.1 cells retain the differentiation capacity of primary airway basal cells

**Figure 4:**
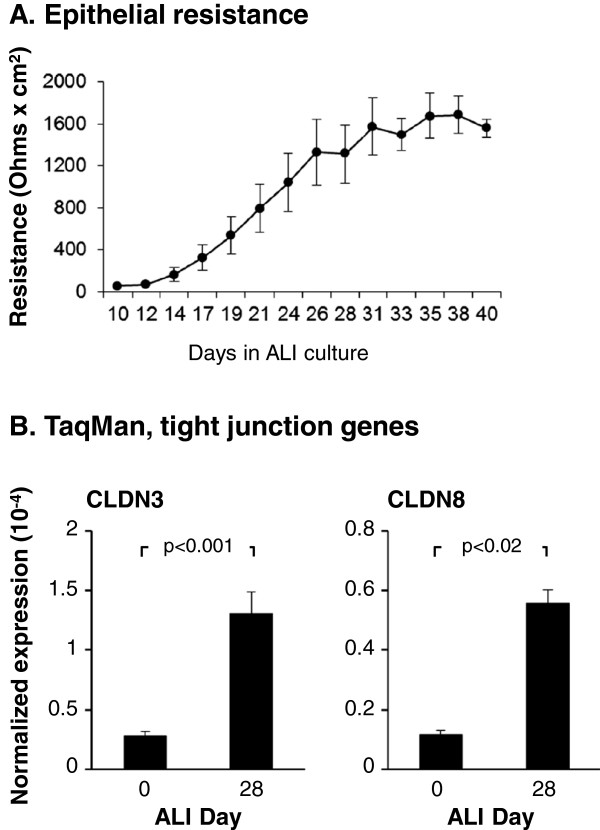
**Tight junction formation of immortalized BCi-NS1.1 cells during air-liquid interface culture. A**. Transepithelial electric resistance (TER) of BCi-NS1.1 cells cultured on air-liquid interface (ALI). Resistance (Ohms x cm^2^) was measured at every media change. Data shown is the average TER ± the standard error of n = 8 independent experiments from passage 6–30 cells between day 10 to 40 of ALI culture. **B**. Expression of tight junction-related genes of BCi-NS1.1 cells. At ALI day 0 and day 28, TaqMan quantitative PCR analysis was used to assess expression of the tight junction related genes claudin 3 (CLDN3) and claudin 8 (CLDN8). Data shown is the average ± the standard error of n = 10 independent experiments for BC-NS1.1 cells from passage 6 to 43. Statistics were calculated by a 2-tailed Student’s t test.

As the progenitor cells of the human airway epithelium, primary BC can be cultured *in vitro* under differentiating air-liquid interface (ALI) culture conditions to produce a fully differentiated mucociliated epithelium [4]. To assess whether BCi-NS1.1 cells retained the progenitor capacity of the primary BC, immortalized BCi-NS1.1 cells were cultured under differentiating ALI conditions for 40 days and the transepithelial electrical resistance (TER), a measure of tight junction formation, was measured at each media change from ALI day 10–40 (Figure 4A). The results demonstrated an increase in TER between ALI day 10 (57 ± 4 Ω × cm^2^) and ALI day 14 (165 ± 67 Ω × cm^2^) which continued to increase for the next 14 days until reaching plateau between ALI day 28 (1312 ± 281 Ω × cm^2^) and ALI day 40 (1563 ± 86 Ω × cm^2^). The levels of TER detected between ALI day 28 to 40 were consistent with those typical of differentiated primary airway epithelial cells [53,54]. To further characterize the ability of BCi-NS1.1 cells to establish tight junctions at the molecular level, we assessed the expression of the tight junction complex genes claudin 3 (CLDN3) and claudin 8 (CLDN8) by TaqMan quantitative PCR during ALI culture. Comparison of ALI day 0 *vs* day 28 demonstrated a significant increase in expression of both genes at ALI day 28 (CLDN3, p < 0.001 and CLDN8, p < 0.02; Figure 4B). These data further strengthen the transepithelial resistance data and support the conclusion that the BCi-NS1.1 cells are capable of polarizing and forming tight junctions during ALI culture.

**Figure 5:**
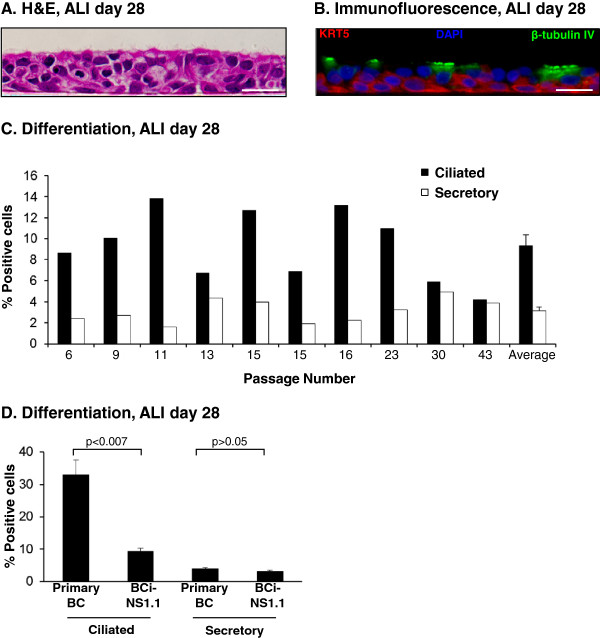
**Differentiation capacity of immortalized BCi-NS1.1 cells over long-term culture. A**. Morphology of ALI day 28 of BCi-NS1.1 cells, passage 30 demonstrating a multilayer ciliated epithelium, hematoxylin and eosin staining **B**. Immunofluorescent staining of ALI day 28 of BCi-NS1.1 cells, passage 30, for KRT5 (basal cell, red), β-tubulin IV (ciliated cell, green) and nuclei (blue). **A**, **B**. Bar = 20 μm. **C**. Quantification of differentiation of ALI day 28 cross-sections of BCi-NS1.1 cells. The number of positive ciliated and secretory cells was scored for BCi-NS1.1 cells for n = 10 independent experiments from cells between passage 6 to 43. The data is presented for each cell type as percentage relative to the total number of cells. In addition, the average ± the standard error of n = 10 independent experiments is shown. **D**. Comparison of the differentiation capacity of BCi-NS1 cells to that of primary airway BC. Primary BC were cultured under identical differentiation-inducing conditions on ALI to that of BCi-NS1.1 cells and quantified in an identical manner. The number of positive ciliated and secretory cells was scored for primary BC and presented for each cell type as percentage relative to the total number of cells. The average ± the standard error of n = 5 independent experiments is shown and compared to the average data for BCi-NS1.1 described in panel C. Statistics were calculated by a 2-tailed Student’s t test.

Differentiation capacity was then confirmed at the histological level by analyzing ALI day 28 cross-sections of BCi-NS1.1 cells which revealed the presence of a pseudostratified ciliated epithelium (Figure 5A). Immunofluorescent staining of the same cross-sections for KRT5 (basal cell marker) and β-tubulin IV (ciliated cell marker) demonstrated positive staining for both basal and ciliated cells at ALI day 28 (Figure 5B). Quantification of BCi-NS1.1 differentiation into a mucociliated epithelium over long-term culture was assessed via Alcian blue staining of ALI day 28 cross-sections generated from BCi-NS1.1 cells ranging from passage 6 to 43. The results demonstrated that, on average, 9.3% ciliated cells and 3.1% Alcian blue positive secretory cells were present at ALI day 28 (Figure 5C). Quantification of KRT5 positive cells in adjacent ALI day 28 cross-sections generated from the same samples by immunofluorescent staining demonstrated an average of 64.6 ± 2.2% of the total cells remained positive for the BC specific marker, suggesting a significant proportion of the BCi-NS1.1 cells did not differentiate (not shown).

The percentage of secretory cells remained relatively stable with increasing passage number of BCi-NS1.1 cells; however, for ciliated cells, there appeared to be a reduction in the capacity of the BCi-NS1.1 cells to differentiate into this cell type from passage 30 onwards (Figure 5C). The reduction in capacity to differentiate into ciliated cells may correspond with the increased presence of cells containing genetic abnormalities, as demonstrated with the karyotype analysis at passage 35 compared to passage 9; however, the differentiation results are obtained from single independent experiments at each passage and therefore, additional experiments are required to confirm this observation and whether or not the increase in genetic instability is the determining factor. To compare the differentiation capacity of primary BC and BCi-NS1.1 cells, nonsmoker primary BC were cultured on ALI for 28 days and differentiation into a mucociliated epithelium assessed as described above for BCi-NS1.1 cells. The results demonstrated a significant decrease in the number of ciliated cells for the BCi-NS1.1 cells *vs* primary BC cultures (9.3 *vs* 32.9%, p < 0.007); however, there were no significant differences in the number of Alcian blue positive secretory cells in the BCi-NS1.1 cells *vs* primary BC cultures (3.1 *vs* 4.0%, p > 0.05; Figure 5D). Overall, these data demonstrate that immortalized BCi-NS1.1 cells are capable of establishing tight junctions and differentiating into a pseudostratified mucociliated epithelium following long-term ALI culture.

### Multipotent differentiation capacity of BCi-NS1.1 and primary airway basal cells

**Figure 6:**
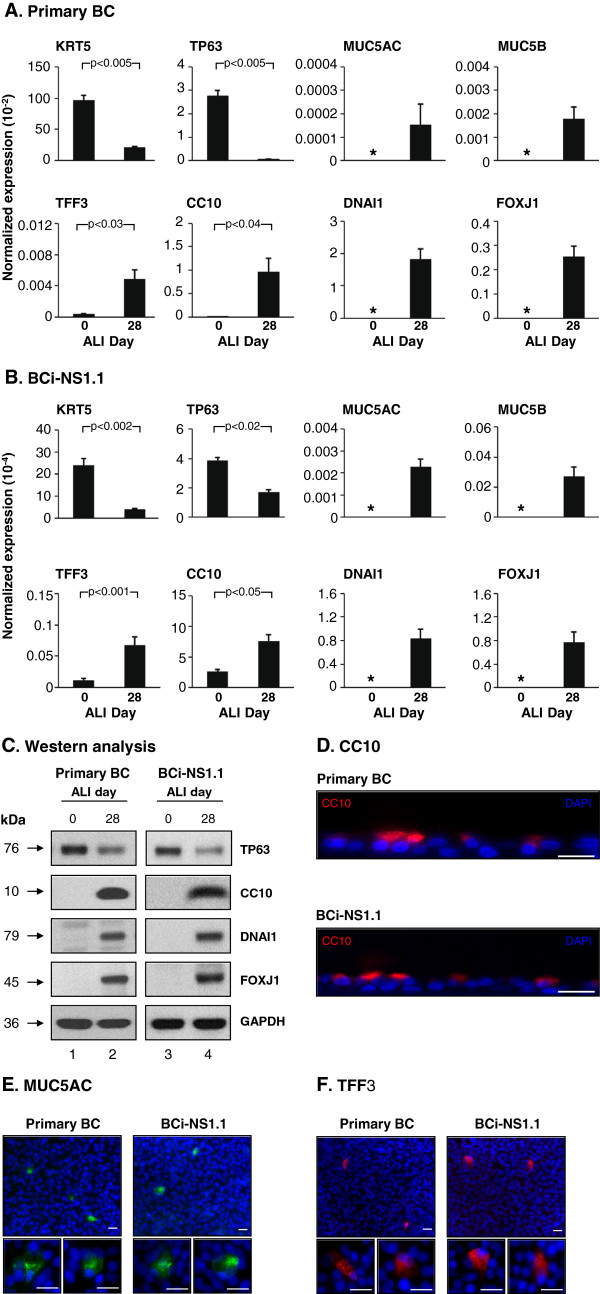
**Comparison of the differentiation capacity of immortalized BCi-NS1 cells to that of primary basal cells.** Primary airway BC and immortalized BCi-NS1 cells were cultured under differentiation inducing conditions on air-liquid interface (ALI). **A, B**. mRNA transcripts; **A**. Primary BC. **B**. BCi-NS1.1 cells. At ALI day 0 and day 28, TaqMan quantitative PCR analysis was used to assess cell type specific mRNA markers: KRT5 (basal cell); TP63 (basal cell); MUC5AC (secretory cell); MUC5B (secretory cell); TFF3 (goblet cell); CC10 (Clara cell); DNAI1 (ciliated cell) and FOXJ1 (ciliated cell). Data shown is the average ± the standard error of n = 5 independent experiments for primary BC and n = 10 independent experiments for BC-NS1.1 cells. Statistics were calculated by a 2-tailed Student’s t test. Asterisk (*) indicates not detected. For **B**, the data represents an average ± the standard error for BCi-NS1.1 cells from passage 6 to 43. **C**. Characterization of differentiation by Western analysis of cell type specific markers. Lane 1 – Primary BC, ALI day 0; lane 2 – Primary BC, ALI day 28; lane 3 – BCi-NS1.1, ALI day 0; and lane 4 – BCi-NS1.1, ALI day 28. Shown is the expression of a basal cell marker (TP63); Clara cell marker (CC10); and ciliated cell markers (DNAI1 and FOXJ1). GAPDH was used as a loading control. **D,E,F**. Immunofluorescent staining of ALI day 28 primary BC and BCi-NS1.1 cells for **D**. CC10 (Clara cell, red), **E**. MUC5AC (secretory cell, green) or **F**. TFF3 (goblet cell, red) and nuclei (blue). Bar = 20 μm. For **C**-**F** all data shown is representative of n = 3 independent experiments.

To further characterize the differentiation capacity of the BCi-NS1.1 cells during ALI culture compared with that observed with primary BC, TaqMan quantitative PCR, Western analysis and immunofluorescent staining using cell type specific markers were undertaken. TaqMan analysis of the primary BC using cell type specific transcript primer-probesets demonstrated multipotent differentiation capacity of the cells (Figure 6A). Comparison of ALI day 0 *vs* day 28 for expression of the BC markers KRT5 and TP63 revealed a significant decrease in expression of each gene at day 28 compared to day 0 (KRT5, p < 0.005 and TP63, p < 0.005), suggesting differentiation of the BC into additional cell types. In contrast, secretory cell markers (MUC5AC and MUC5B) and ciliated cell markers (DNAI1 and FOXJ1) were negative at ALI day 0 but readily detected at ALI day 28, confirming differentiation of the BC into the secretory and ciliated cell types. Analysis of both TFF3 (goblet cell marker) and CC10 (Clara cell marker) revealed a low level of expression at ALI day 0; however, a significant increase in expression was observed at ALI day 28 (TFF3, p < 0.03; CC10, p < 0.04), demonstrating the primary BC can differentiate into both the goblet and Clara cell types.

Analysis of the BCi-NS1.1 cells cultured under identical conditions to that of the primary BC demonstrated similar results (Figure 6B). As observed for the primary BC, comparison of ALI day 0 *vs* day 28 from BCi-NS1.1 cells for expression of the BC markers KRT5 and TP63 revealed a significant decrease in expression of each gene at day 28 compared to day 0 (KRT5, p < 0.002 and TP63, p < 0.02). Similarly to primary BC, secretory cell markers (MUC5AC and MUC5B) and ciliated cell markers (DNAI1 and FOXJ1) were negative at ALI day 0 but readily detected at ALI day 28, confirming differentiation of the BCi-NS1.1 cells into the secretory and ciliated cell types. For both TFF3 (goblet cell marker) and CC10 (Clara cell marker), low levels of expression at ALI day 0 were detected; however, like primary BC, a significant increase in expression was observed at ALI day 28 (TFF3, p < 0.001; CC10, p < 0.05), demonstrating BCi-NS1.1 cells can differentiate into both goblet and Clara cells.

Western analysis of primary BC comparing day 0 (Figure 6C, lane 1) and day 28 (Figure 6C, lane 2) demonstrated expression of the basal cell marker TP63 at both time points, with a reduction in levels at ALI Day 28, while Clara (CC10) and ciliated (DNAI1, FOXJ1) cell markers were only detected at ALI day 28 (Figure 6C). Analysis of BCi-NS1.1 cells revealed identical expression patterns at day 0 (lane 3) and day 28 (lane 4) for the same markers. Immunofluorescent staining of CC10 (Clara), MUC5AC (secretory) and TFF3 (goblet) revealed positivity for all these cells types in ALI day 28 cultures of primary BC and BCi-NS1.1 cells (Figure 6D-F).

Overall, these data demonstrate that both primary BC and immortalized BCi-NS1.1 cells have multipotent differentiation capacity and are capable of differentiating into the secretory, goblet, Clara and ciliated cell types during *in vitro* ALI culture.

### IL-13 modulation of differentiation of BCi-NS1.1 cells

**Figure 7:**
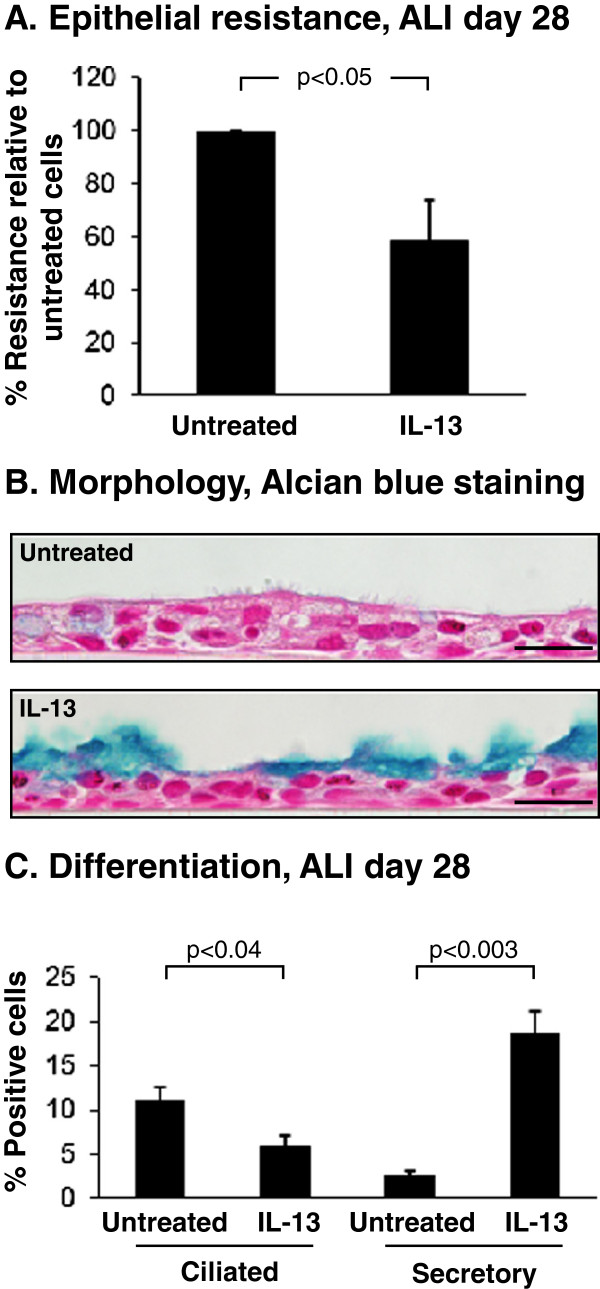
**IL-13 modulation of differentiation of BCi-NS1.1 immortalized airway basal cells.** BCi-NS1.1 cells were cultured under differentiation inducing conditions on air-liquid interface (ALI) in the absence and presence of IL-13. **A**. Transepithelial electric resistance (TER) of the airway epithelia in the absence and presence of IL-13. The resistance (Ohms x cm^2^) was measured at ALI day 28. Data is presented as the percentage resistance relative to untreated cells and plotted as the average ± the standard error of n = 5 independent experiments (BCi-NS1.1, passage 6–15). **B**. Morphology of untreated and IL-13 treated cells. Alcian blue staining of ALI day 28 sections of BCi-NS1.1 cells untreated or treated with IL-13. Bar = 20 μm. **C**. Quantification of differentiation. Shown is Alcian blue staining of ALI day 28 sections of BCi-NS1.1 cells untreated or treated with IL-13. The number of positive ciliated and secretory cells scored. Data is presented for each cell type as percentage relative to the total number of cells. Data shown is the average ± the standard error of n = 5 independent experiments (BCi-NS1.1, passage 6–15). All statistics were calculated by a 2-tailed Student’s t test.

Dysregulation of the progenitor function of primary airway BC by cytokine exposure plays an important role in mediating the pathology associated with respiratory diseases characterized by airway remodeling, including asthma and COPD [8,26,28-30]. One such cytokine is interleukin-13 (IL-13), a known mediator of regulating mucus metaplasia and mucin production, and capable of increasing secretory cell differentiation of primary normal human bronchial epithelium *in vitro*[53,55,56]. BCi-NS1.1 cells were cultured on ALI and at ALI day 10, exposed to IL-13 until harvest at ALI day 28 for analysis of differentiation. Measurement of the TER at ALI day 28 demonstrated a significant decrease in epithelial resistance in IL-13 treated cells relative to untreated cells (41.4% decrease, p < 0.05; Figure 7A), suggesting disordering of BCi-NS1.1 differentiation. This was confirmed at the histological level via Alcian blue staining of ALI day 28 cross-sections of untreated and IL-13 stimulated BCi-NS1.1 cells (Figure 7B). Quantification of these changes demonstrated a significant decrease in the number of ciliated cells for the IL-13 treated *vs* untreated cultures (10.9 *vs* 5.8%, p < 0.04) with a reciprocal increased number of Alcian blue positive secretory cells in the IL-13 treated *vs* untreated cultures (2.6 *vs* 18.5%, p < 0.003; Figure 7C).

**Figure 8:**
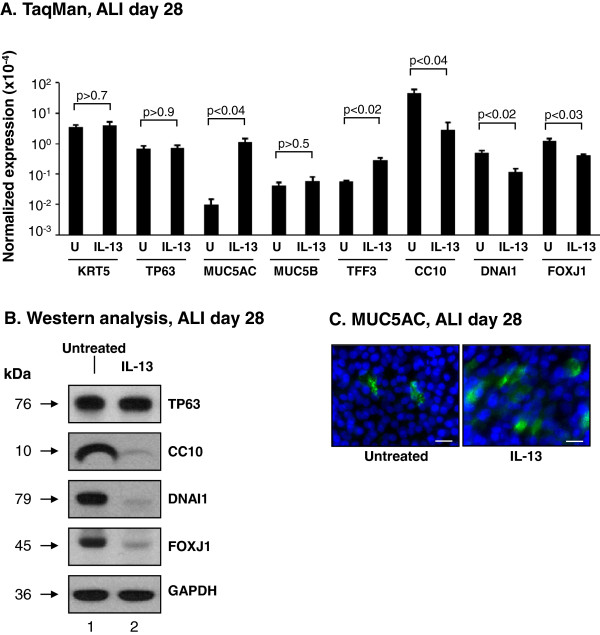
**Quantification of IL-13 modulation of the differentiation of BCi-NS1.1 immortalized airway basal cells.** BCi-NS1.1 cells were cultured on air-liquid interface (ALI) in the absence and presence of IL-13. **A**. TaqMan analysis of cell type specific mRNA markers at ALI day 28, including basal cell markers (KRT5, TP63); secretory cell markers (MUC5AC, MUC5B); goblet cell marker (TFF3); Clara cell marker (CC10); and ciliated cell markers (DNAI1, FOXJ1). Data shown is the average ± the standard error of n = 5 independent experiments (BCi-NS1.1, passage 6–15). Statistics were calculated by 2-tailed Student’s t test. U = untreated; IL-13 = treated with IL-13. **B**. Western analysis of untreated and IL-13 treated cells of cell type specific markers at ALI day 28. Lane 1 – untreated, lane 2 – IL-13 treated. Shown is the expression of a basal cell marker (TP63); Clara cell marker (CC10) and ciliated cell markers (DNAI1, FOXJ1). GAPDH was used as a loading control. **C**. Immunofluorescent staining of ALI day 28 membranes for untreated and IL-13 treated cells for MUC5AC (secretory cell, green) and nuclei (blue). Bar = 20 μm. For **B** and **C**, all data shown is representative of n = 3 independent experiments.

To further characterize the effects of IL-13 on the differentiation capacity of the BCi-NS1.1 cells, TaqMan quantitative PCR, Western analysis and immunofluorescent staining using cell type specific markers were assessed. TaqMan analysis of ALI day 28 untreated *vs* IL-13 stimulated cells with cell type specific transcript primer-probesets demonstrated significant differences in expression of a subset of cell type specific markers (Figure 8A). Analysis of the BC markers KRT5 (p > 0.7) and TP63 (p > 0.9) revealed no significant changes in expression upon stimulation with IL-13. For the secretory cell marker MUC5AC (p < 0.04) and goblet cell marker TFF3 (p < 0.02) there was a significant increase in expression upon IL-13 stimulation, but no significant changes were observed for the secretory cell marker MUC5B (p > 0.5). In contrast to MUC5AC and TFF3, there was a significant decrease in expression of the Clara cell marker CC10 (p < 0.04) and ciliated cell markers DNAI1 (p < 0.02) and FOXJ1 (p < 0.03) in IL-13 stimulated *vs* untreated cells. These data were validated at the protein level by Western analysis and immunofluorescent staining. Western analysis of untreated (Figure 8B, lane 1) and IL-13 stimulated (lane 2) ALI day 28 BCi-NS1.1 cells demonstrated equal expression of the basal cell markers KRT5 and TP63. However, levels of Clara (CC10) and ciliated (DNAI1 and FOXJ1) cell markers were decreased in the IL-13 stimulated cells. Immunofluorescent staining of MUC5AC (secretory) revealed increased percentage of positive cells in IL-13 stimulated *vs* untreated BCi-NS1.1 cells (Figure 8C). Overall, these data demonstrate that the differentiation potential of ALI cultures of BCi-NS1.1 cells can be altered by exposure to specific stimuli known to play a role in mediating the pathology of respiratory diseases.

### BCi-NS1.1 cell retain the capacity to repair injured areas of the epithelium

**Figure 9:**
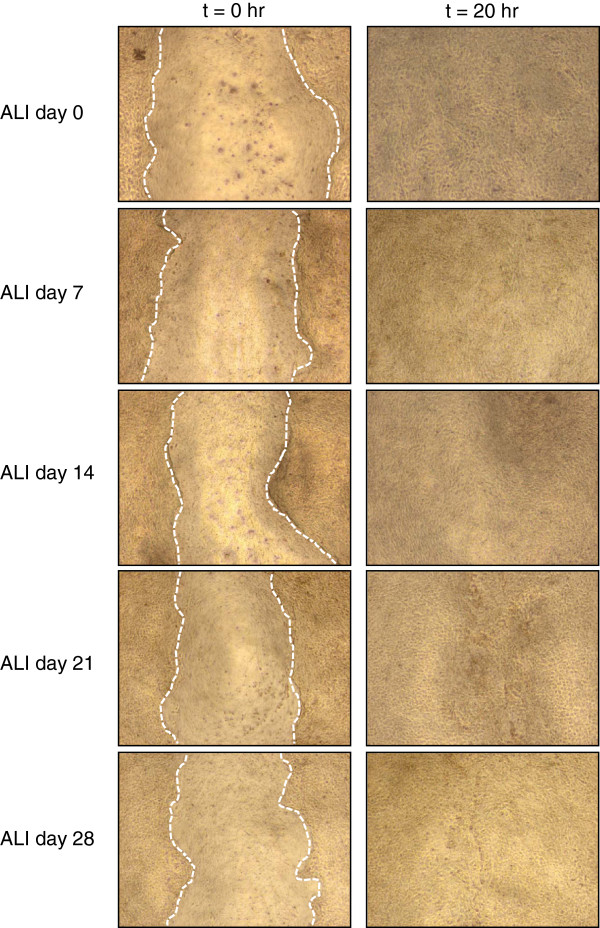
**Wound healing capacity of immortalized BCi-NS1.1 cells.** BCi-NS1.1 cells were cultured on air-liquid interface (ALI) culture and the ability of the cells to repair injury to the epithelium assessed at ALI day 0, 7, 14, 21 and 28. Images were obtained at the time of injury (t = 0) and 20 hr later (t = 20) once repair had taken place. All data shown is representative of n = 5 independent experiments using BCi-NS1.1 cells between passage 7 to 44.

We next assessed the ability of the BCi-NS1.1 cells to respond to mechanical damage during ALI culture by performing simple scratch and wound healing assays (Figure 9). The results demonstrated that BCi-NS1.1 cells were capable of repairing the wounded area within 20 hr when assessed at all ALI time points (day 0, 7, 14, 21 and 28), suggesting that repair processes, including the required synthesis and secretion of basal lamina matrices, are maintained with immortalization. Comparable results were obtained with the parental immortalized BCi-NS1 cells at early time points (ALI day 0 and 7); however, around ALI day 10–14, the cells spontaneously began to leak and lose integrity which prevented their analysis at later time points (not shown).

## Discussion

Basal cells function as multipotent stem/progenitor cells of the mouse and human airway epithelium, capable of differentiating into the specialized cell populations of the mucociliated airway epithelium [4-18]. The focus of this study was to establish an immortalized normal human airway BC cell line capable of replicating long-term and retaining characteristics of primary BC. Primary human airway BC isolated from the airway epithelium of a healthy nonsmoker were successfully immortalized via retrovirus-mediated expression of hTERT to create the cell line basal cell immortalized-nonsmoker 1 (BCi-NS1). A clonal derived population (BCi-NS1.1) retains characteristics of primary BC for over 40 passages including secretion of VEGFA, interaction with endothelial cells, ability to actively repair wounded areas of the epithelium and multipotent differentiation capacity on air-liquid interface (ALI) culture with differentiation into secretory, goblet, Clara and ciliated cells. Importantly, these cells respond to external stimuli such as IL-13, resulting in alteration of the normal differentiation process.

Primary human airway BC are positive for the pan-BC markers KRT5 and TP63, with subpopulations that demonstrate positivity for additional markers including KRT14, CD151, NGFR and ITGA6 [4-7,11]. The clonal immortalized BCi-NS1.1 cell line was positive for KRT5, TP63 and CD151. When placed on ALI on type IV collagen, BCi-NS1.1 cells were capable of establishing high levels of transepithelial electrical resistance, and like primary BC, differentiated into all cell types of the conducting airway. In addition, stimulation of BCi-NS1.1 cells during ALI culture with IL-13, a cytokine known to play an important role in the pathology of asthma and COPD *in vivo*, resulted in alteration of the normal differentiation response with stimulated cells displaying increased secretory cell differentiation, and a corresponding decrease in Clara and ciliated cell differentiation consistent with previous studies using cultures of primary normal bronchial epithelial cells [53,55,56]. The resulting alteration in BCi-NS1.1 differentiation produce a remodeled *in vitro* pseudostratified epithelium reminiscent of that observed *in vivo* in asthma and COPD [8,26,28-30], demonstrating the potential use of this cell line in understanding the mechanisms by which environmental stimuli play a central role in the pathology of clinically relevant airway disease.

Long term cultures of immortalized normal human bronchial epithelium have previously been generated from bronchial-derived donor material using a number of different methods, including adenovirus-SV40 hybrid virus; plasmid containing replication defective SV40 virus genome; plasmid or retroviral gene transfer-mediated delivery of viral oncoproteins (HPV-16 E6 and E7, or SV40 T-antigen) alone or together with hTERT, or expression of hTERT alone or in combination with CDK4 or Bmi-1 (Additional file 1: Table S1). These established cell lines have an extended lifespan far beyond the primary cells from which they were derived and retain some capacity to differentiate into other specialized cells of the airway epithelium. For example, BEAS-2B cells, which were developed by immortalization of normal human bronchial epithelial cells using an adenovirus-SV40 hybrid virus, retain the capacity for squamous differentiation, while 16HBE14o- cells (bronchial epithelium immortalized with plasmid containing a replication deficient SV40 virus genome) can differentiate into ciliated cells in ALI culture [35,41,57]. Similarly, immortalizing bronchial epithelial cells using HPV-16 E6 and E7 alone (VA10 cells) or together with hTERT (NuLi 1–2) resulted in cell lines capable of differentiating into ciliated (VA10) or ciliated and goblet cells (NuLi-2) on ALI culture [39,40,58]. Combined expression of SV40 early antigen and hTERT resulted in immortalization of small airway epithelial cells to generate the cell line SA (SV40 ER + hTERT) which, when injected subcutaneously into a nude mouse, produced airway epithelium with normal histology [38]. The alternative strategy of immortalization in the absence of viral oncoproteins resulted in the generation of cell lines with differentiation capacity. Combined expression of hTERT and CDK4, allowed for the generation of the HBEC3 cell line that differentiates into a mucociliated epithelium during ALI when cultured on a fibroblast containing collagen gel [42]. Submerged 2D culture of these cells in defined differentiation media resulted in simultaneous expression of both basal, Clara and type II pneumocyte markers [59-61]. A similar strategy using combined expression of hTERT and Bmi-1 resulted in generation of the UNCN1T, 2 T and 3 T cell lines that retained the ability to differentiate into a pseudostratified epithelium with ciliated and secretory cells [46].

The differences in differentiation capacity observed between the cell lines may be explained by a number of factors, including the immortalization strategy utilized and the culture conditions used to analyze differentiation. The different origins and source of primary cells used in each study may be an equally important factor. In the present study, the primary BC donor material for establishing the immortalized BCi-NS1.1 cells was obtained by bronchial brushing of a healthy nonsmoker, whereas the donor material used in previous studies was from bronchial derived explants; autopsy specimens; lung transplants; bronchial epithelium; or commercially available sources (Additional file 1: Table S1). Characterization of the HBEC3 and VA10 cell lines demonstrate they express BC markers (HBEC3: TP63^+^KRT14^+^ and VA10: TP63^+^KRT5^+^KRT14^+^), suggesting the primary cells from which they were derived were of BC origin [40,61]. In addition to the potential role of the source of donor material on differentiation capacity of the generated immortalized cell lines, it may also have a significant impact on the efficiency of different strategies used to immortalize the cells. For instance, Ramirez et al. [42] demonstrated that both hTERT and CDK4 were required to immortalize airway epithelial cells. However, our results and those of Piao et al. [43] demonstrated that hTERT alone was sufficient. The use of different donor materials between each study may result in different sub-populations of primary epithelial cells at the time of immortalization which may have different requirements for immortalization.

Recent studies have demonstrated that primary human epithelial cells (including human airway epithelium) can be propagated indefinitely *in vitro* when co-cultured with irradiated fibroblast feeder cells and a Rho kinase inhibitor [33,34]. These cultured cells are termed conditionally reprogrammed cells (CRCs), as removal of the reprogramming conditions allows the cells to differentiate in a tissue-specific manner. One major advantage of this system over generating immortalized cell lines is that the CRC’s are karyotype stable, thus allowing cell biology studies without the potential negative effects of using genetically unstable cell cultures. However, the more complex culture system, potential variability between clinical donor material, and expensive costs of acquiring primary cells may prevent rapid adoption of this technology. In the context of these factors, human bronchial epithelium-derived cell lines provide a viable alternative.

## Conclusion

Basal cells have a centrmental insult, including smoking and inflammatory cytokines [4-18]. Importantly, airway BC contribute to the pathogenesis of diseases characterized by airway epithelial remodeling, including COPD and asthma, and the biology of the malignant transformation of the epithelium into bronchogenic carcinoma [8,19-30]. The BCi-NS1.1 cells, together with other immortalized BC cell lines, provide cell populations to help understand the mechanisms by which the differentiation capacity of the BC is regulated, and how the cells respond to specific environmental stimuli is central to the understanding of these diseases. Finally, the ability to isolate primary BC from individuals by bronchoscopic brushing and subsequently immortalize them provides a strategy to deriving patient specific BC cell lines that can be used to study the effect of specific genetic variants on BC differentiation under normal conditions or in response to specific environmental stimuli, including cytokines or cigarette smoke.

The BCi-NS1.1 cell line is available for use by investigators (geneticmedicine@med.cornell.edu).

## Competing interests

The authors declare that they have no competing interests.

## Authors’ contributions

MSW conceived of the study and its design, performed research, data analysis and interpretation, and manuscript writing. KG performed research, and data analysis and interpretation. BA performed research, and data analysis and interpretation. MASM performed data analysis and interpretation. VA performed research. JH performed research. BSD performed research, and data analysis and interpretation. SR performed data analysis and interpretation. MRS performed research, and data analysis and interpretation. RGC conceived of the study and its design, performed data analysis and interpretation, manuscript writing, and gave final approval of manuscript. All authors read and approved the final manuscript.

## Supplementary Material

Additional file 1 **Table S1.** Summary of immortalized human airway epithelial cell lines. **Figure S1.** Cell viability analysis of parental and clonal immortalized BCi-NS1 cells over continuous serial passage. **Figure S2.** Growth rate analysis of immortalized BCi-NS1.1 following extended serial passage. Click here for file
