# Supplementary material for: Generation of a human airway epithelium derived basal cell line with multipotent differentiation capacity
Source: Respir Res. 2013 Dec 3;14(1):135. doi: 10.1186/1465-9921-14-135 (PMC3907041; doi:10.1186/1465-9921-14-135)
Supplement: Additional file 1 — Table S1. Summary of immortalized human airway epithelial cell lines. Figure S1. Cell viability analysis of parental and clonal immortalized BCi-NS1 cells over continuous serial passage. Figure S2. Growth rate analysis of immortalized BCi-NS1.1 following extended serial passage. [file 1465-9921-14-135-S1.pdf]

Supplemental Table I. Summary of Immortalized Human Airway Epithelial Cell Lines[1]

Name	Immortalization method	Donor material	Differentiation conditions	Differentiation capacity	References
BEAS-2B	Hybrid adeno-SV40 virus	Bronchial epithelium from explants of autopsy specimens from noncancerous individuals	Submerged culture	Squamous differentiation	1
			ALI[2] culture	No ciliated or secretory cells	2
HTEo-	Origin defective SV40 genome	Enzymatically isolated tracheal cells or outgrowths of tracheal tissue explants	Not reported	Not reported	3
16HBE14o-	Origin defective SV40 genome	Bronchial surface epithelial cells from a 1yr-old male heart-lung transplant patient	ALI culture with vitrogen coated support and serum containing media	Ciliated cells	4
hTBE	SV40 early region and hTERT	Tracheal and bronchial epithelium from lung transplants	Not reported	Not reported	5
SA (SV40 ER + hTERT)	SV40 early region and hTERT	"Small airway epithelial cells" expressing cytokeratin 19 from commercial source[3]	Subcutaneous injection of nude mouse	Histology similar to normal airway epithelium	5
NuLi-1-2	HPV-16 E6/E7 and hTERT	Bronchial epithelium enzymatically isolated from healthy donor lungs	ALI culture with collagen coated support and ultroser G containing media	NuLi-2 differentiated into goblet and ciliated cells	6
HBEC1-4	CDK4 and hTERT	Bronchial epithelium from areas of the lung histologically not involved with lung cancer	ALI culture with IMR90 fibroblast-contracted collagen gel	HBEC3 cells differentiated into goblet and ciliated cells	7-9
			3D matrigel co-culture with IMR90 fibroblasts	HBEC3 cells produce spheroid cyst-like structures	10
			2D submerged monolayer culture for 2 days in differentiation media	HBEC3 cells simultaneously express markers for basal, Clara and type II pneumocytes	10

Supplemental Table I. Summary of Immortalized Human Airway Epithelial Cell Lines (cont. page 2)

Name	Immortalization method	Donor material	Differentiation conditions	Differentiation capacity	References
AE-hTERT (F and M)	hTERT	Passage 2 - small airway epithelial cells from one female and one male nonsmoker from commercial source[3]	Not reported	Not reported	11
VA10	HPV-16 E6/E7	Primary bronchial epithelial cells	ALI culture with collagen coated support and ultroser G containing media	Ciliated cells	12,13
			3D matrigel co-culture model with HUVECs[4]	Produce 3D bronchioalveolar like structures	14
UNCN1T-3T	Bmi-1 and hTERT	Human bronchial epithelial cells cultured from lung tissue obtained from male and female donors	ALI culture with collagen coated support	All three cell lines differentiated into secretory and ciliated cells	15

[1] Cell lines generated using only normal tissue as primary donor material.
[2] Air-liquid interface.
[3] Bio-Whittaker/Clonetics (Walkersville, MD).
[4] Human umbilical cord vein endothelial cells.

Supplemental References

1. Reddel RR, Ke Y, Gerwin BI, McMenamin MG, Lechner JF, Su RT, Brash DE, Park JB, Rhim JS, Harris CC: **Transformation of human bronchial epithelial cells by infection with SV40 or adenovirus-12 SV40 hybrid virus, or transfection via strontium phosphate coprecipitation with a plasmid containing SV40 early region genes.** *Cancer Res* 1988, **48:**1904-1909.

2. Stewart CE, Torr EE, Mohd Jamili NH, Bosquillon C, Sayers I: **Evaluation of differentiated human bronchial epithelial cell culture systems for asthma research.** *J Allergy (Cairo)* 2012, **2012:**943982.

3. Gruenert DC, Basbaum CB, Welsh MJ, Li M, Finkbeiner WE, Nadel JA: **Characterization of human tracheal epithelial cells transformed by an origin-defective simian virus 40.** *Proc Natl Acad Sci U S A* 1988, **85:**5951-5955.

4. Cozens AL, Yezzi MJ, Kunzelmann K, Ohrui T, Chin L, Eng K, Finkbeiner WE, Widdicombe JH, Gruenert DC: **CFTR expression and chloride secretion in polarized immortal human bronchial epithelial cells.** *Am J Respir Cell Mol Biol* 1994, **10:**38-47.

5. Lundberg AS, Randell SH, Stewart SA, Elenbaas B, Hartwell KA, Brooks MW, Fleming MD, Olsen JC, Miller SW, Weinberg RA et al.: **Immortalization and transformation of primary human airway epithelial cells by gene transfer.** *Oncogene* 2002, **21:**4577-4586.

6. Zabner J, Karp P, Seiler M, Phillips SL, Mitchell CJ, Saavedra M, Welsh M, Klingelhutz AJ: **Development of cystic fibrosis and noncystic fibrosis airway cell lines.** *Am J Physiol Lung Cell Mol Physiol* 2003, **284:**L844-L854.

7. Ramirez RD, Sheridan S, Girard L, Sato M, Kim Y, Pollack J, Peyton M, Zou Y, Kurie JM, Dimaio JM et al.: **Immortalization of human bronchial epithelial cells in the absence of viral oncoproteins.** *Cancer Res* 2004, **64:**9027-9034.

8. Sato M, Vaughan MB, Girard L, Peyton M, Lee W, Shames DS, Ramirez RD, Sunaga N, Gazdar AF, Shay JW et al.: **Multiple oncogenic changes (K-RAS(V12), p53 knockdown, mutant EGFRs, p16 bypass, telomerase) are not sufficient to confer a full malignant phenotype on human bronchial epithelial cells.** *Cancer Res* 2006, **66:**2116-2128.

9. Vaughan MB, Ramirez RD, Wright WE, Minna JD, Shay JW: **A three-dimensional model of differentiation of immortalized human bronchial epithelial cells.** *Differentiation* 2006, **74:**141-148.

10. Delgado O, Kaisani AA, Spinola M, Xie XJ, Batten KG, Minna JD, Wright WE, Shay JW: **Multipotent capacity of immortalized human bronchial epithelial cells.** *PLoS One* 2011, **6:**e22023.

11. Piao CQ, Liu L, Zhao YL, Balajee AS, Suzuki M, Hei TK: **Immortalization of human small airway epithelial cells by ectopic expression of telomerase.** *Carcinogenesis* 2005, **26:**725-731.

12. Benediktsdottir BE, Arason AJ, Halldorsson S, Gudjonsson T, Masson M, Baldursson O: **Drug delivery characteristics of the progenitor bronchial epithelial cell line VA10.** *Pharm Res* 2013, **30:**781-791.

13. Halldorsson S, Asgrimsson V, Axelsson I, Gudmundsson GH, Steinarsdottir M, Baldursson O, Gudjonsson T: **Differentiation potential of a basal epithelial cell line established from human bronchial explant.** *In Vitro Cell Dev Biol Anim* 2007, **43:**283-289.

14. Franzdottir SR, Axelsson IT, Arason AJ, Baldursson O, Gudjonsson T, Magnusson MK: **Airway branching morphogenesis in three dimensional culture.** *Respir Res* 2010, **11:**162.

15. Fulcher ML, Gabriel SE, Olsen JC, Tatreau JR, Gentzsch M, Livanos E, Saavedra MT, Salmon P, Randell SH: **Novel human bronchial epithelial cell lines for cystic fibrosis research.** *Am J Physiol Lung Cell Mol Physiol* 2009, **296:**L82-L91.

Supplemental Figure Legends

Supplemental Figure 1. Cell viability analysis of parental and clonal immortalized BCi-NS1 cells over continuous serial passage. For both parental and clonal BCi-NS1.1 cell lines, cell viability was assessed using trypan blue exclusion following trypsinization during early and late passages. **A.** Parental BCi-NS1 cells were assessed at eleven early passages (passage 8-20) and eleven later passages (passage 35-46). **B.** Clonal BCi-NS1.1 cells were assessed at twelve early passages (passage 6-23) and twelve later passages (passage 41-71). For both **A.** and **B.,** data shown is the average ± the standard error. Statistics were calculated by 2-tailed Student's t test.

Supplemental Figure 2. Growth rate analysis of immortalized BCi-NS1.1 following extended serial passage. BCi-NS1.1 cells from early (passage 9-12); middle (passage 46-49) and late (passage 71-74) passages were seeded in 12-well plates at a density of 3000 cells/cm^2 in growth media in a side by side comparison and harvested every 24 hr to assess the growth rate of the cells. Data shown is the average ± the standard error of n=4 independent experiments. Statistics were calculated by 2-tailed Student's t test.

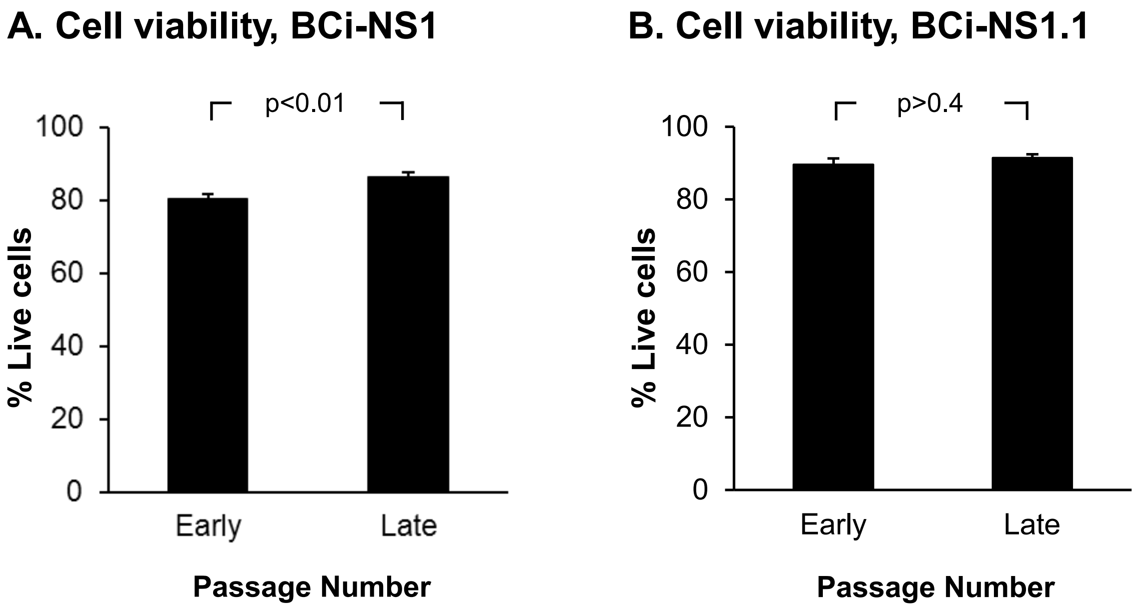
A. Cell viability, BCi-NS1
p<0.01
100
80
60
40
20
0
% Live cells
Early
Late
Passage Number
B. Cell viability, BCi-NS1.1
p>0.4
100
80
60
40
20
0
% Live cells
Early
Late
Passage Number

Figure S2

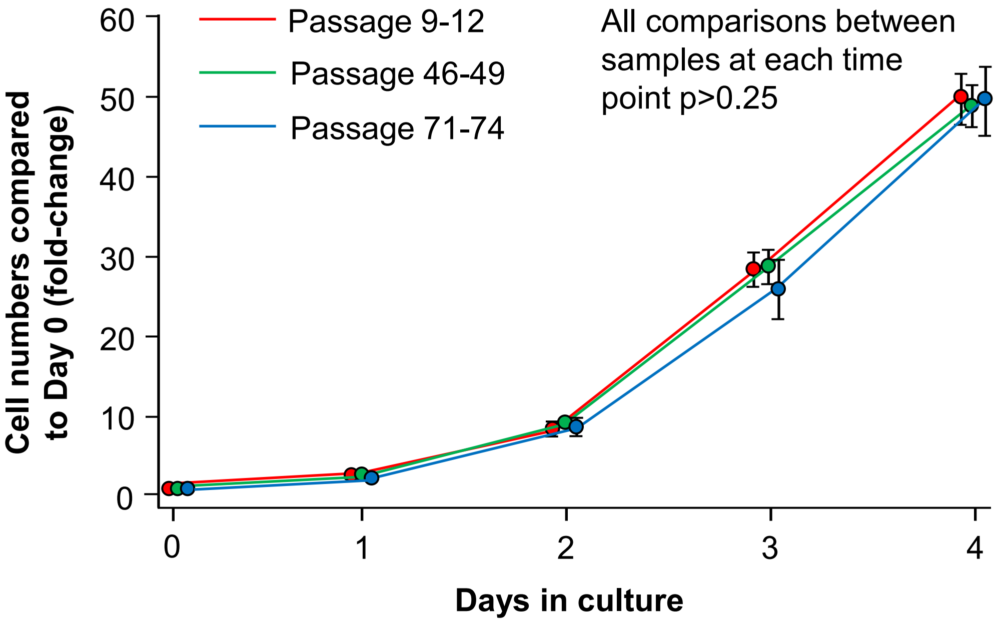
Passage 9-12
Passage 46-49
Passage 71-74
All comparisons between samples at each time point p>0.25
Cell numbers compared to Day 0 (fold-change)
0
10
20
30
40
50
60
0
1
2
3
4
Days in culture
